# LncRNA DANCR promotes the proliferation, migration, and invasion of tongue squamous cell carcinoma cells through miR-135a-5p/KLF8 axis

**DOI:** 10.1186/s12935-019-1016-6

**Published:** 2019-11-19

**Authors:** Ying Zheng, Bowen Zheng, Xue Meng, Yuwen Yan, Jia He, Yi Liu

**Affiliations:** 1 Department of Orthodontics, School of Stomatology, China Medical University, 117 North Nanjing Street, Shenyang, 110002 People’s Republic of China; 2 Department of Stomatology, Shengjing Hospital of China Medical University, Shenyang, 110004 People’s Republic of China

**Keywords:** DANCR, Tongue squamous cell carcinoma, miR-135a-5p, KLF8, MMP

## Abstract

**Background:**

Tongue squamous cell carcinoma (TSCC) is a most invasive cancer with high mortality and poor prognosis. It is reported that lncRNA DANCR has implications in multiple types of cancers. However, its biological role and underlying mechanism in TSCC progress are not well elucidated.

**Methods:**

Our present study first investigated the function of DANCR on the proliferation, migration and invasion of TSCC cells by silencing or overexpressing DANCR. Further, the miR-135a-5p-Kruppel-like Factor 8 (KLF8) axis was focused on to explore the regulatory mechanism of DANCR on TSCC cell malignant phenotypes. Xenografted tumor growth using nude mice was performed to examine the role of DANCR in vivo.

**Results:**

DANCR knockdown reduced the viability and inhibited the migration and invasion of TSCC cells in vitro, while ectopic expression of DANCR induced opposite effects. In vivo, the tumor growth and the expression of matrix metalloproteinase (MMP)-2/9 and KLF8 were also blocked by DANCR inhibition. In addition, we found that miR-135-5p directly targeted DANCR, which was negatively correlated with DANCR on TSCC progression. Its inhibition reversed the beneficial effects of DANCR silence on TSCC malignancies. Furthermore, the expression of KLF8 evidently altered by both DANCR and miR-135a-5p. Silencing KLF8 using its specific siRNA showed that KLF8 was responsible for the induction of miR-135a-5p inhibitor on TSCC cell malignancies and MMP-2/9 expression.

**Conclusions:**

These findings, for the first time, suggest that DANCR plays an oncogenic role in TSCC progression via targeting miR-135a-5p/KLF8 axis, which provides a promising biomarker and treatment approach for preventing TSCC.

## Background

Tongue squamous cell carcinoma (TSCC) is a major type of head and neck squamous cell carcinoma (HNSCC) with high recurrence rates, increased proliferation and metastasis, and poor prognosis [1, 2]. Despite of significant advances in the prevention and treatment, the survival rates of TSCC patients are still low [3]. It is identified that the invasion and migration mainly contribute to the progression of tumors. Therefore, it is urgent that developing novel therapeutic strategies for TSCC through the exploration of the underlying molecular mechanisms.

LncRNAs are a group of long non-coding RNAs with more than 200 nucleotides in length. Numerous reports has shown that lncRNAs play important roles in wide ranges of biological processes, including cell proliferation, differentiation, apoptosis, migration and invasion [4–6]. Especially, multiple lncRNAs has been found to be closely implicated in the tumorigenesis and progression of TSCC. For example, high-expression of lncRNA AFAP1-AS1 in TSCC tumor tissues enhances tumor progression via the activation of Wnt/β-catenin signaling pathway [7]. NKILA serves as a crucial determinant of TSCC metastasis to reduce the migratory and invasive cells through inhibiting the process of epithelial–mesenchymal transition (EMT) [8]. Interestingly, lncRNA DANCR (differentiation antagonizing non-protein coding RNA) has been noticed to suppress epidermal cell differentiation [9] and improve hepatocellular carcinoma self-renewal [10]. DANCR is also taken as an oncogenic lncRNA for several cancers, such as prostate cancer [11], gastric cancer [12] and colorectal cancer [13]. However, the distinct function of DANCR in TSCC was not well understood.

MicroRNAs (miRNAs), a class of small non-coding RNAs, are shown to modulate the expression of target genes. Recent studies have revealed that miR-135a-5p is the main regulator of tumor invasion and metastasis [14, 15]. In non-small cell lung cancer (NSCLC), miR-135a-5p is demonstrated to inhibit cell migration and invasion through targeting Kruppel-like Factor 8 (KLF8) [16]. As we know, KLF8 has been widely confirmed to participate in the regulation of cell cycle progression, transformation, EMT and invasion [17–21]. Given that DANCR was predicted to have putative binding sites with miR-135a-5p through the analysis of online bioinformatics, we thus speculated that DANCR might affect the development and progression of TSCC by regulating miR-135a-5p/KLF8 axis.

To improve the understanding of DANCR effects on TSCC malignancies, CAL-27 and TCa-8113 cells with DANCR silence, and SCC9 and TSCCA cells with DANCR overexpression were constructed. Then the effects of DANCR on the proliferation, migration and invasion of TSCC cells were determined. Further, miR-135a-5p/KLF8 axis was focused to explore the molecular mechanism by which DANCR promoted TSCC progression.

## Methods

### Cell culture and reagents

In our experiments, four human TSCC cell lines (SCC9, TSCCA, TCa-8113 and CAL-27 cells) were used. SCC9 cells (Cellcook, Guangzhou, China) were cultured in DMEM/F12 medium supplemented with 10% fetal bovine serum (FBS; SH30084.03, Hyclone, South Logan, UT, USA); TSCCA cells (Procell, Wuhan, China) were maintained in DMEM medium (12100-46, Gibco) containing with 10% FBS; TCa-8113 and CAL-27 cell lines (Procell, Wuhan, China) were cultured in RPMI-1640 medium (31800-014, Gibco, Gaithersburg, MD, USA) supplemented with 10% FBS. All these cell lines were cultured in a standard environment at 37 °C with 5% CO_2_. MiR-135a-5p mimics/inhibitor and corresponding negative control (NC) mimics/inhibitor were purchased from JTS Scientific (Beijing, China).

### Construction of siRNAs and shRNAs

The sequences of siRNAs (5′–3′) targeting human DANCR were designed as follows: si-DANCR-1 sense GUUGACAACUACAGGCACATT and antisense UGUGCCUGUAGUUGUCAACTT; si-DANCR-2 sense CUAGAGCAGUGACAAUGCUTT and antisense AGCAUUGUCACUGCUCUAGTT. The NC siRNA sequences (5′–3′) were: sense UUCUCCGAACGUGUCACGUTT and antisense ACGUGACACGUUCGGAGAATT. Then shRNAs targeting DANCR and corresponding NC were constructed by pRNAH1.1 plasmid vectors (Genscript, Nanjing, China).

Furthermore, we also designed the interfering sequences (5′–3′) for human KLF8 as follows: si-KLF8 sense CGAUAUGGAUAAACUCAUATT and antisense UAUGAGUUUAUCCAUAUCGAC. The corresponding NC siRNA sequences (5′–3′) were designed as follows: si-NC sense UUCUCCGAACGUGUCACGUTT and antisense ACGUGACACGUUCGGAGAATT.

### Construction of overexpression plasmids

A pair of specific primers (forward 5′-CAAGGATCCGCCCTTGCCCAGAGTCTTCC-3′ and reverse 5′-CCGCTCGAGGTCAGGCCAAGTAAGTTTAT-3′) was used to amplify human DANCR (NR_024031.2). Then the amplified products were inserted into pcDNA3.1 plasmids (V790-20, Invitrogen, Carlsbad, CA, USA) between BamHI and XhoI restriction enzyme sites to induce the overexpression of DANCR. The empty pcDNA3.1 vector was used as control.

### Cell transfection

When cells reached at 70% of confluence, siRNAs or shRNAs targeting DANCR were transfected into CAL-27 and TCa-8113 cells, and ectopic expression of DANCR were transfected into SCC9 and TSCCA cells by the mediation of Lipofectamine 2000 reagent (11668-019, Invitrogen) following the manufacturer’s instructions. All experiments were performed at 48 h post transfection.

In addition, miR-135a-5p mimics or NC mimics was transfected into CAL-27 or TCa-8113 cells, and its inhibitor or NC inhibitor was transfected into SCC9 cells as mentioned above to overexpress or silence miR-135a-5p. Furthermore, the co-transfection of miR-135a-5p inhibitor and si-DANCR or si-KLF8 was also mediated by Lipofectamine 2000.

### Quantitative real-time polymerase chain reaction (qRT-PCR)

Total RNAs in TSCC cell lines were extracted with RNAsimple Total RNA Kit (DP419, TIANGEN, Beijing, China) and reverse-transcribed into cDNA templates using M-MLV reverse transcriptase (NG212, TIANGEN). The designed specific primer sequences were synthesized by Sangon Biotech (Shanghai, China) and shown as follows (5′–3′): miR-135a-5p, RT GTTGGCTCTGGTGCAGGGTCCGAGGTATTCGCACCAGAGCCAACTCACAT, forward GCCGTATGGCTTTTTATTCCTA and reverse GGTGCAGGGTCCGAGGTATT; U6, RT GTTGGCTCTGGTGCAGGGTCCGAGGTATTCGCACCAGAGCCAACAAAATATGG, forward GCTTCGGCAGCACATATACT and reverse GGTGCAGGGTCCGAGGTATT; DANCR forward ACCCTCCTGCTTCCCTC and reverse CCCGAAACCCGCTACAT; KLF8 forward TCATTGGAGGAGATGGTAA and reverse GCTGCTGGTTCTTGCTGT; GAPDH forward GACCTGACCTGCCGTCTAG and reverse AGGAGTGGGTGTCGCTGT. Subsequently, the mixture of cDNA templates, specific primers, SYBR Green reagent (SY1020, Solarbio, Beijing, China) and Taq PCR MasterMix (KT201, TIANGEN) were used to amplify target genes by qRT-PCR analysis on Exicycler 96 PCR system (Bioneer, Daejeon, Korea). GAPDH was normalized for DANCR and KLF8 expression, and U6 was normalized for miR-135a-5p expression. Relative expression was calculated using the 2^− ΔΔCT^ method.

### MTT assay

TSCC cells were seeded in 96-well plates at the density of 4 × 10^3^ cells/well for 0, 24, 48 or 72 h, respectively. Then cells were incubated in a complete medium containing 0.5 mg/ml MTT (KGA311, KeyGEN, Nanjing, China) for 4 h. After dissolving in DMSO (ST038, Beyotime), the viable cells were determined using microplate reader (ELX-800, BIOTEK, Winooski, VT, USA) at the optical density of 570 nm.

### Wound healing assay

The wound healing assay was used to assess cell migratory ability. Cells were treated with mitomycin C (M0503, Sigma) for 1 h in a serum-free medium. Then a wound scratch was made by a 200 μl pipette tip in the culture plate and recorded it by phase-contrast microscopy (IX53, Olympus, Tokyo, Japan) under 100× magnification. Twenty-four hours later, the migratory distances were measured with Image Pro Plus Software (Media Cybernetics, Silver Springs, MD, USA) to calculate the capacity of cell migration.

### Transwell assay

Transwell assay was utilized to evaluate the invasive ability of cells. Briefly, cell suspensions (2 × 10^4^ cells/well) were seeded in the upper chamber of 24-well Transwell inserts (3422, Corning Incorporated, Corning, NY, USA) pre-coated with Matrigel (356234, BD Biosciences, San Jose, CA, USA) with serum-free medium. The lower chamber was filled with the medium containing with 30% FBS. After 48 h of incubation, cells in the upper chamber were removed and washed in PBS twice. Then cells were fixed in 4% paraformaldehyde and stained with 0.4% crystal violet (0528, Amresco, Solon, OH, USA). The number of cells in the lower chamber was observed by phase-contrast microscope under 200× magnification. Five fields in each image were randomly selected to count and the invasive cell ratio was normalized to control.

### Luciferase reporter assay

Bioinformatics analysis predicted that lncRNA DANCR had putative binding sites with miR-135a-5p. The pmirGLO vector (E133A, Promega, Madison, WI, USA) containing NheI and SalI restriction enzyme sites was applied to construct wild type (wt) or mutant type (mut) luciferase reporter vectors for DANCR. The site-directed mutation of DANCR was used to verify the target effects between DANCR and miR-135a-5p. Then 293T cells (ZhongQiaoXinZhou Bio, Shanghai, China) were seeded in 12-well plates and co-transfected with wt-DANCR, or mut-DANCR together with miR-135a-5p or NC mimics using Lipofectamine 2000. Finally, the binding activity was tested with a dual luciferase reporter assay kit (E1910, Promega) by the calculation of Firefly luciferase activity/Renilla luciferase activity at 48 h post-transfection.

### Western blot

Total proteins from TSCC cell lines or tumor tissues were isolated using RIPA lysate (R0010, Solarbio) containing PMSF (P0100, Solarbio) and quantified using BCA assay kit (PC0020, Solarbio). Then equal proteins were loaded on the Sodium dodecylsulphate polyacrylamide gel electrophoresis (SDS-PAGE) gel, and transferred onto PVDF membrane (IPVH00010, Millipore, Billerica, MA, USA). After washing in TBST, the membrane was incubated with one of the following specific primary antibodies overnight at 4 °C: MMP-2 antibody (1:500; 10373-2-AP, Proteintech, Wuhan, China), MMP-9 antibody (1:500; ab38898, Abcam, Cambridge, UK), KLF8 antibody (1:1000; A16321, Abclonal, Wuhan, China) and GAPDH (1:10,000; 60004-1-Ig, Proteintech). Subsequently, horseradish peroxidase (HRP)-conjugated goat anti-rabbit antibody (1:3000; SE134, Solarbio) or HRP-conjugated goat anti-mouse antibody (1:3000; SE131, Solarbio) was used to incubate with the membrane for 1 h at 37 °C. Protein signals were developed with ECL kit (PE0010, Solarbio) and quantified using Gel-Pro-Analyzer Software (Media Cybernetics, Silver Springs, MD, USA). GAPDH was used as internal control.

### Xenograft tumor model analysis

The ethical approval was obtained from School of Stomatology, China Medical University Committee (No. G2018007) in this study. All animal experimental procedures were performed according to the Guide for the Care and Use of Laboratory Animals. The Balb/c-nude mice (4–5 weeks, 18–20 g) were purchased from HuaFuKang Bioscience Co. lnc (Beijing, China) and housed in a standard environment. Stably transfected cells with sh-DANCR or sh-NC were selected using G418 antibiotics (A1720, Sigma, St. Louis, MO, USA). Then, CAL-27 cells or TCa-8113 cells with sh-DANCR or sh-NC stable transfections were subcutaneously injected into the right side of axilla at the density of 1 × 10^6^ cells per animal. Tumor volume was measured using the caliper every 4 days following the formula: tumor volume (mm^3^) = (length × width^2^)/2. Tumor weight was measured when mice were killed after 25 days.

### Immunofluorescence

For immunofluorescence staining, the collected tumor tissues were fixed in paraformaldehyde, embedded with paraffin and sectioned into 5 μm-thickness slides. Then paraffin slides were incubated with specific primary antibody against KLF8 (NBP2-57740, NOVUS, Centennial, CO, USA) overnight at 4 °C, and conjugated with FITC-labeled goat anti-rabbit secondary antibody (A0562, Beyotime) at room temperature for 60 min. After counterstaining with DAPI, the immunopositive materials were visualized using optical microscope (BX53, Olympus) at the magnification of 400× and captured using digital camera (DP73, Olympus).

### Statistical analysis

Data were expressed as mean ± SD and analyzed using GraphPad Prism software (San Diego, CA, USA). The comparisons were performed using t-test or one-way ANOVA following Bonferroni’s test. p < 0.05 was identified to indicate a significant difference statistically.

## Results

### DANCR knockdown suppressed the proliferation, migration and invasion of TSCC cell lines

**Fig. 1:**
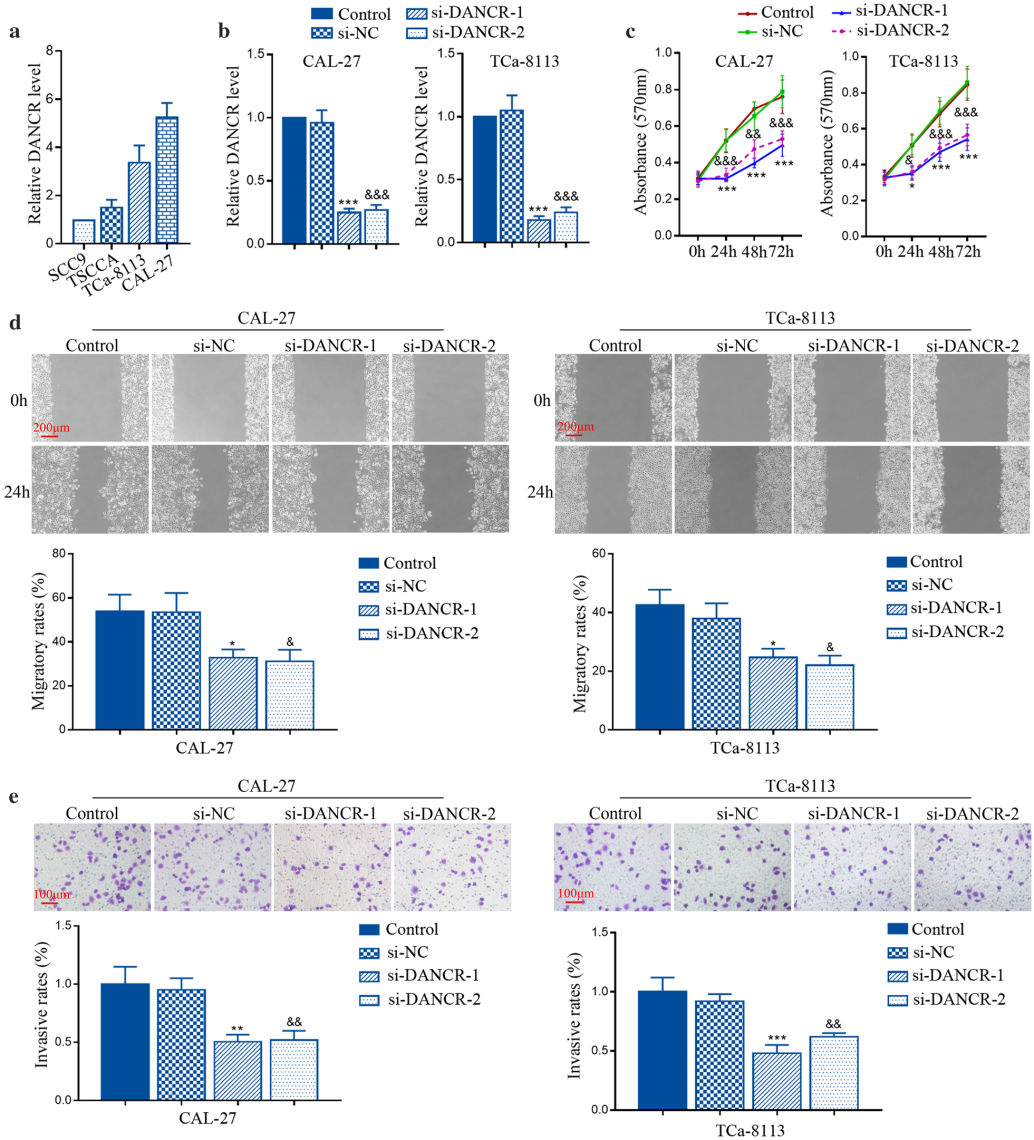
DANCR knockdown suppressed the proliferation, migration and invasion in vitro. **a** Relative expression of DANCR was detected by qRT-PCR in different TSCC cell lines. **b** CAL-27 and TCa-8113 cells were transfected with siRNAs against DANCR. The relative expression of DANCR was detected by qRT-PCR. **c** The viability of CAL-27 and TCa-8113 cells was assessed by MTT assay. **d**, **e** The migration and invasion of CAL-27 and TCa-8113 cells was determined using wound healing assay and transwell assay, respectively. *p < 0.05, **p < 0.01, ***p < 0.001; ^&^p < 0.05, ^&&^p < 0.01, ^&&&^p  < 0.001, versus to si-NC

In four different TSCC cell lines, the expression profile of DANCR was first detected as shown in Fig. 1a. From this chart, it was apparent that DANCR expression was higher in CAL-27 and TCa-8113 cells than in SCC9 and TSCCA cells. Thus in further experiments, CAL-27 and TCa-8113 cells were used to inhibit DANCR, while SCC9 and TSCCA cells were forced to express DANCR. As expectation, specific siRNAs targeting DANCR significantly decreased its levels in CAL-27 and TCa-8113 cells (Fig. 1b).

Then the effects of si-DANCRs on the proliferation, migration and invasion of TSCC cells were first assessed. MTT assay was considered to indicate cell proliferation, and the results showed that DANCR knockdown reduced the viable number of CAL-27 and TCa-8113 cells (Fig. 1c). Furthermore, it seemed that inhibition of DANCR significantly decreased the migratory and invasive ability of TSCC cells using wound healing assay and transwell invasion assay (Fig. 1d, e). These results indicate that DANCR knockdown may attenuate TSCC malignancies in vitro.

### DANCR overexpression promoted the proliferation, migration and invasion of TSCC cell lines

**Fig. 2:**
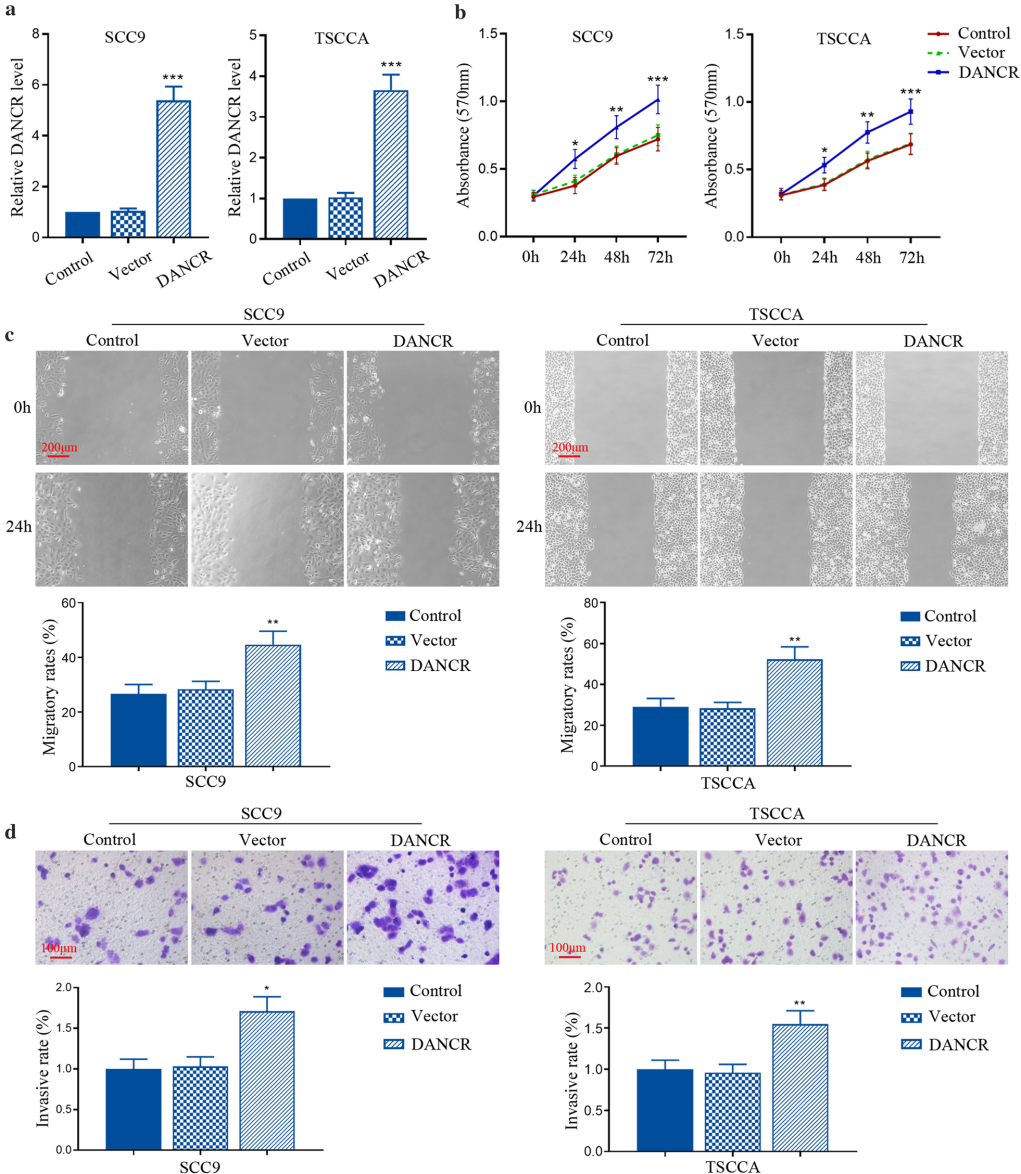
DANCR overexpression promoted the proliferation, migration and invasion in vitro. **a** SCC9 and TSCCA cells were transfected with pcDNA3.1 vector expressing DANCR. The relative expression of DANCR was detected by qRT-PCR. **b** The viability of SCC9 and TSCCA cells was assessed by MTT assay. **c**, **d** The migration and invasion of SCC9 and TSCCA cells was determined using wound healing assay and transwell assay, respectively. *p < 0.05, **p < 0.01, ***p < 0.001, versus to vector

Further, the forced expression of DANCR was used to investigate its biological function in SCC9 and TSCCA cells. We observed a marked increase of DANCR expression by its overexpression plasmids in SCC9 and TSCCA cells (Fig. 2a). Functional analysis from SCC9 and TSCCA cells indicated that the ectopic expression of DANCR induced increments of cell viability, migratory distance and invasive cell number (Fig. 2b–d). Our data show that DANCR can enhance the proliferation, migration and invasion of TSCC cells in vitro.

### DANCR targeted miR-135a-5p to regulate KLF8 expression in TSCC cell lines

**Fig. 3:**
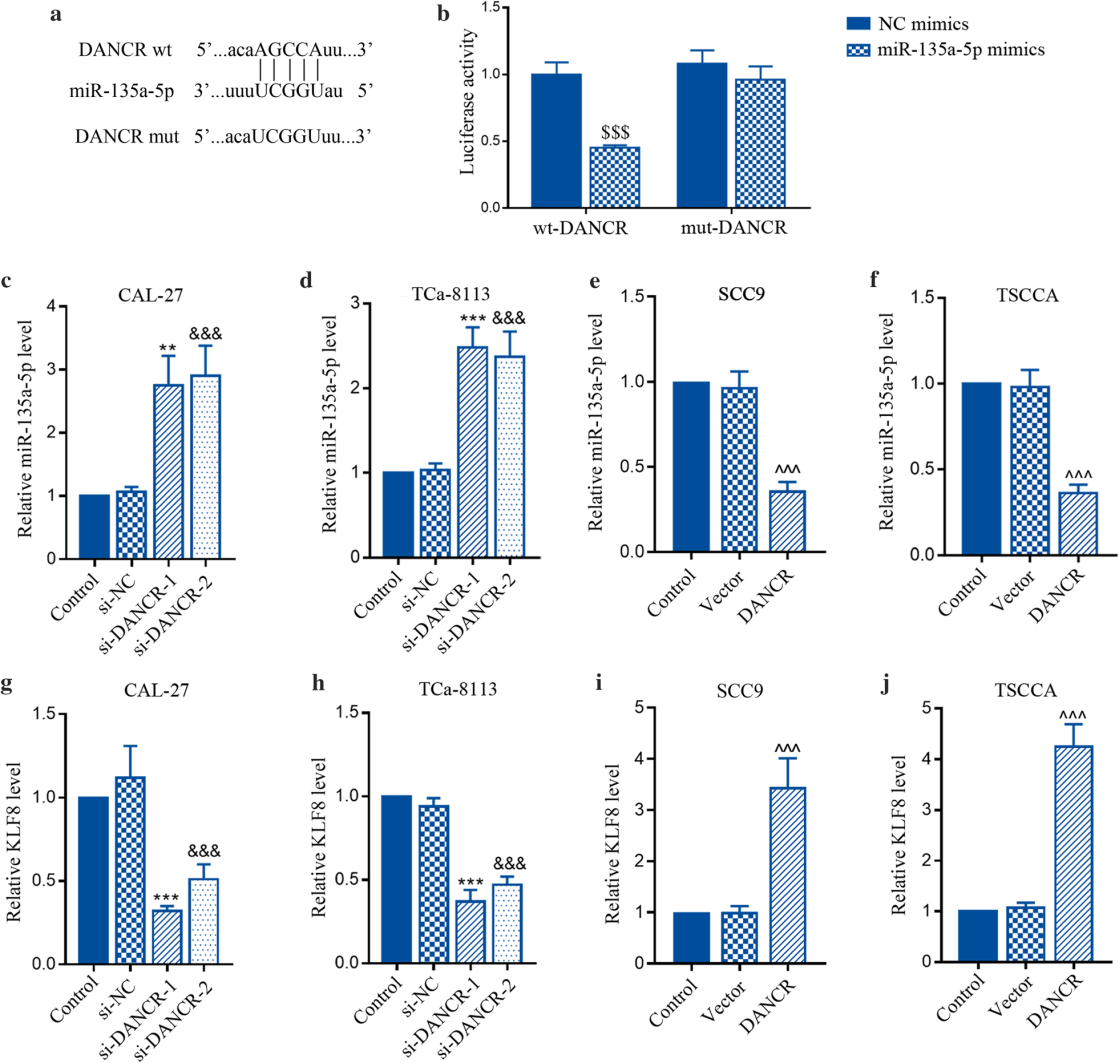
DANCR targeted miR-135a-5p to regulate KLF8 expression in vitro. **a** Sequence alignments of DANCR with potential targeting sites of miR-135a-5p. **b** Luciferase reporter assay was performed to verify the binding effect between DANCR and miR-135a-5p. **c**–**f** Relative expression of miR-135a-5p was examined by qRT-PCR in CAL-27 cells (**c**), TCa-8113 cells (**d**), SCC9 cells (**e**) and TSCCA cells (**f**). **g**–**j** Relative expression of KLF8 was detected using qRT-PCR in CAL-27 cells (**g**), TCa-8113 cells (**h**), SCC9 cells (**i**) and TSCCA cells (**j**). ^$$$^p < 0.001, versus to wt-DANCR + NC mimics. **p < 0.01, ***p < 0.001; ^&&&^p < 0.001, versus to si-NC. ^^^^^p < 0.001, versus to Vector

As shown in Fig. 3a, the bioinformatics predicted that DANCR was complementary with miR-135a-5p (Fig. 3a), which was confirmed by dual luciferase reporter assay. The results demonstrated that miR-135a-5p mimics significantly inhibited the luciferase activity of wt-DANCR, but not mut-DANCR (Fig. 3b). Then we observed a marked increase of miR-135a-5p level in CAL-27 and TCa-8113 cells transfected with si-DANCR (Fig. 3c, d), but a significant reduction of miR-135a-5p in SCC9 and TSCCA cells transfected with pcDNA3.1-DANCR (Fig. 3e, f). In addition, KLF8 mRNA was down-expressed by knockdown of DANCR in CAL-27 (Fig. 3g) and TCa-8113 cells (Fig. 3h), but increased by DANCR overexpression in SCC9 (Fig. 3i) and TSCCA cells (Fig. 3j). These data suggest that miR-135a-5p is a direct target of DANCR, and KLF8 may participate in DANCR-mediated regulation of TSCC malignant phenotypes.

### MiR-135a-5p overexpression suppressed tumor cell progression and KLF8 expression in TSCC cell lines

**Fig. 4:**
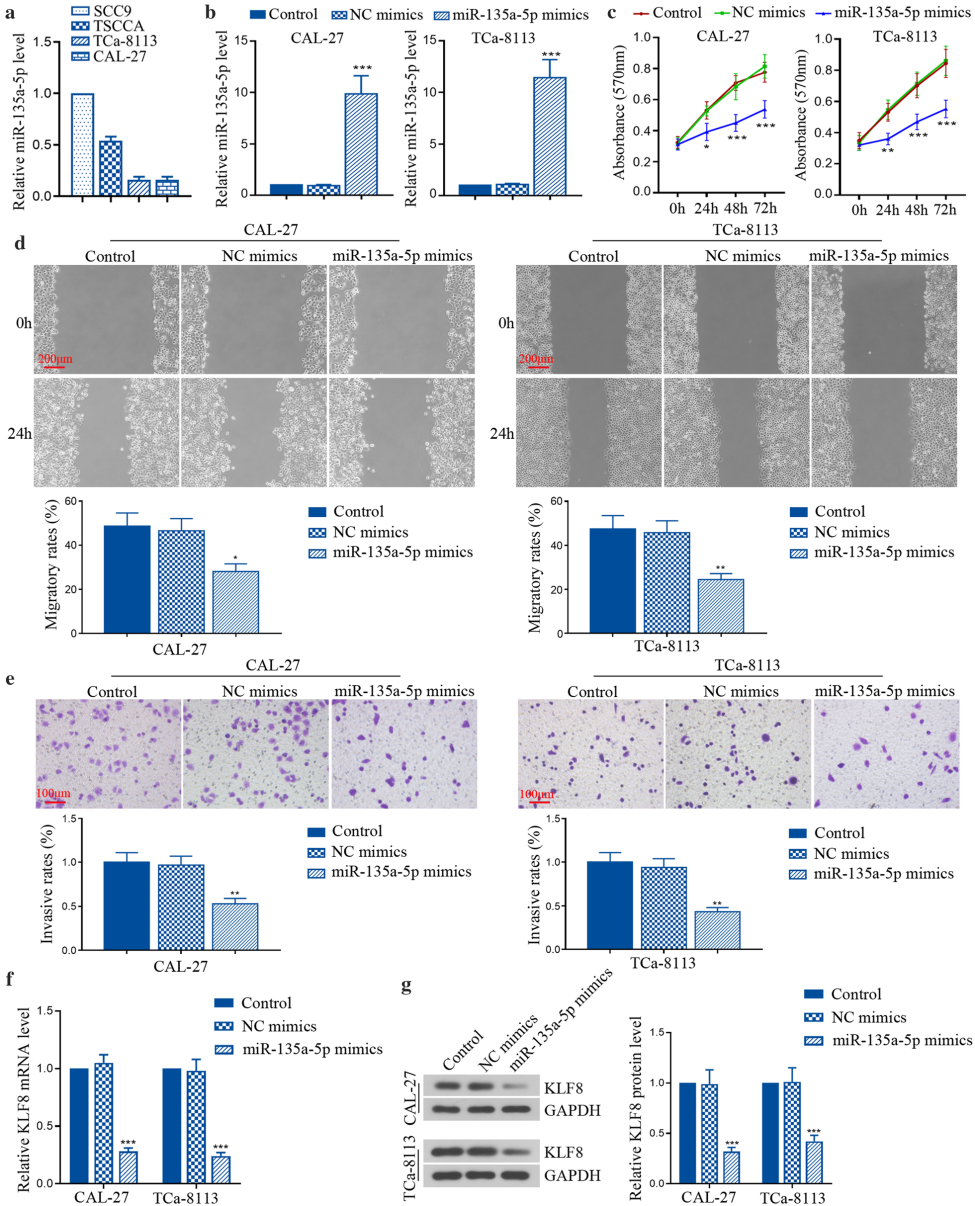
MiR-135a-5p overexpression suppressed tumor cell progression and KLF8 expression in vitro. **a** Relative expression of miR-135a-5p in different TSCC cell lines was examined using qRT-PCR. **b** Relative expression of miR-135a-5p was measured in CAL-27 and TCa-8113 cells transfected with miR-135a-5p mimics by qRT-PCR. **c** The viability of CAL-27 and TCa-8113 cells was measured using MTT assay. **d**, **e** The migration and invasion of CAL-27 and TCa-8113 cells was examined using wound healing assay and transwell assay, respectively. **f** Relative expression of KLF8 mRNA was detected in CAL-27 and TCa-8113 cells using qRT-PCR. **g** Relative expression of KLF8 protein was measured using western blot in CAL-27 and TCa-8113 cells. *p < 0.05, **p  < 0.01, ***p < 0.001, versus to NC mimics

Then we found that miR-135a-5p expression in SCC9 and TSCCA cells was higher than that in TCa-8113 and CAL-27 cells (Fig. 4a). To further investigate the role of miR-135a-5p, its specific mimics were further carried out. It obviously confirmed that miR-135a-5p expression was increased by its mimics in CAL-27 and TCa-8113 cells (Fig. 4b). The results in Fig. 4c–e showed that overexpression of miR-135a-5p reduced viable cells, shortened migratory distance and decreased invasive cells in CAL-27 cells and TCa-8113 cells. In addition, KLF8 mRNA and protein expression were also suppressed by miR-135a-5p (Fig. 4f, g). All results indicate that miR-135a-5p may protect against TSCC malignant phenotypes with the involvement of KLF8 suppression.

### DANCR knockdown repressed tumor cell progression and KLF8 expression by targeting miR-135a-5p in TSCC cell lines

**Fig. 5:**
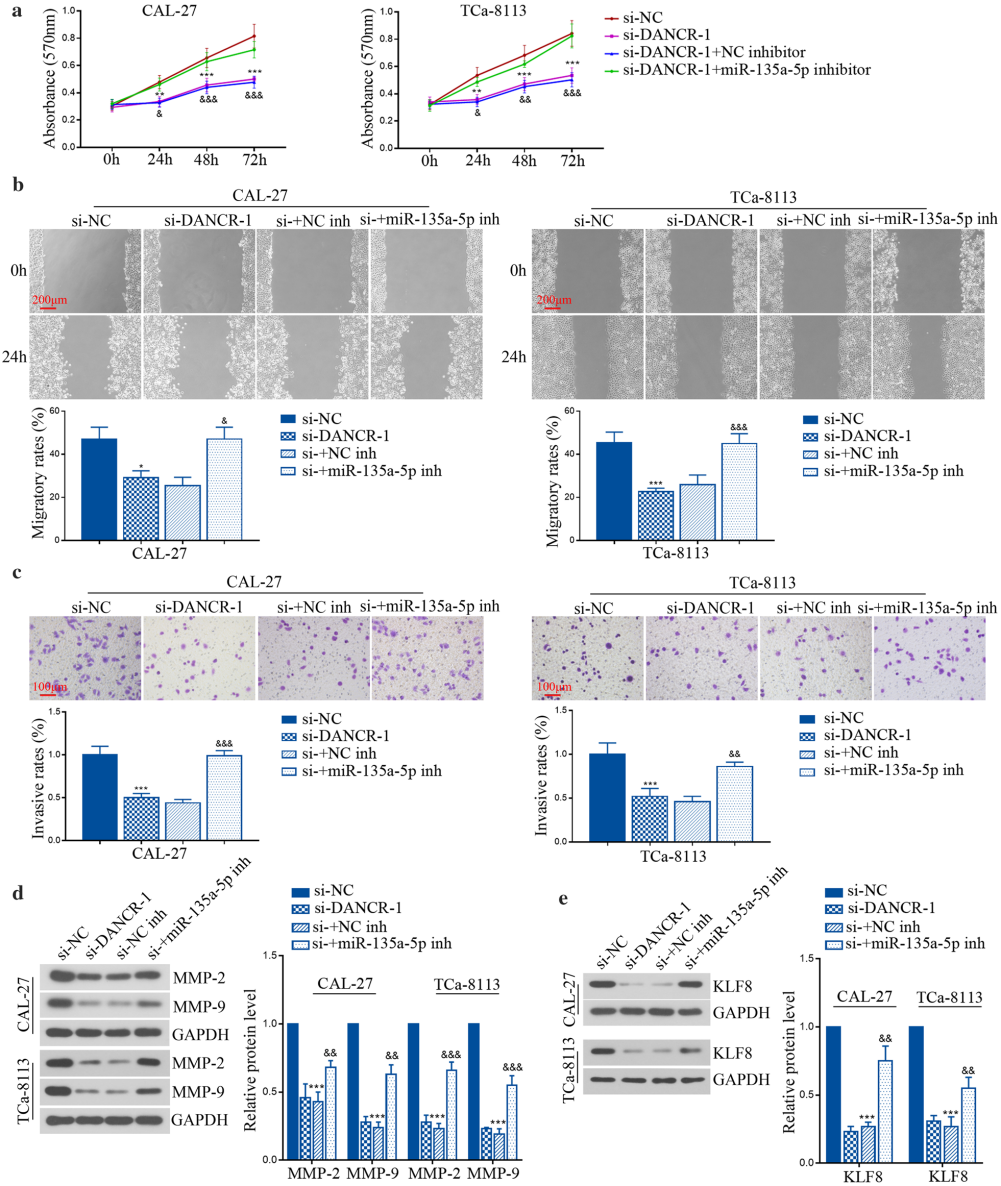
DANCR knockdown repressed tumor cell progression and KLF8 expression by targeting miR-135a-5p in vitro. **a** The viability of CAL-27 and TCa-8113 cells was measured using MTT assay. **b**, **c** The migration and invasion of CAL-27 and TCa-8113 cells were detected by wound healing assay and transwell assay, respectively. **d** Relative expression of MMP-2 and MMP-9 protein was determined by western blot in CAL-27 and TCa-8113 cells. **e** Relative expression of KLF8 protein was detected using western blot in CAL-27 and TCa-8113 cells. *p < 0.05, **p < 0.01, ***p < 0.001, versus to si-NC; ^&^p < 0.05, ^&&^p < 0.01, ^&&&^p < 0.001, versus to si-DANCR-1 + NC inhibitor

Although miR-135a-5p had been identified to target DANCR and be beneficial for TSCC progress, whether miR-135a-5p was responsible for the effects of DANCR on tumor malignancies was unclear. As illustrated in Fig. 5a, the reduction of viable cells by DANCR knockdown was enhanced by miR-135a-5p inhibitor. Furthermore, inhibition of miR-135a-5p reversed si-DANCR-mediated suppression of cell migration and invasion (Fig. 5b, c). It is well-known that matrix metalloproteinase (MMP) family proteins are main biomarkers for tumor invasion and metastasis. Results in Fig. 5d showed that the decrease of MMP-2 and MMP-9 protein levels induced by DANCR silence was partially increased by miR-135a-5p inhibitor. In addition, we found that the reduction of KLF8 in si-DANCR cells was increased by miR-135a-5p inhibitor (Fig. 5e). Together the results further suggest that DANCR/miR-135a-5p may modulate TSCC progression by the regulation of KLF8.

### MiR-135a-5p inhibition exacerbated tumor cell progression through activating KLF8 in TSCC cell lines

**Fig. 6:**
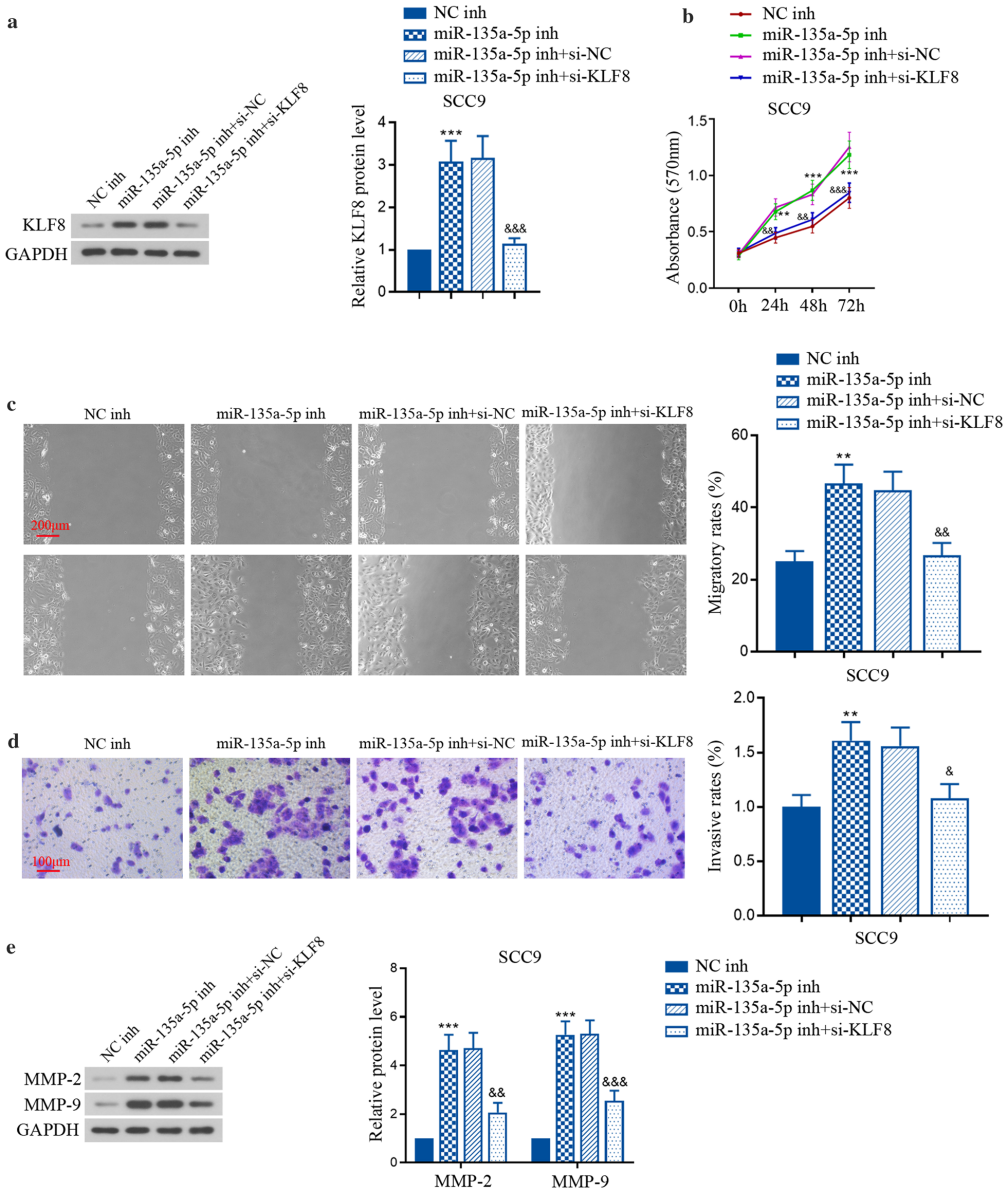
MiR-135a-5p inhibition exacerbated tumor cell progression through activating KLF8 in vitro. **a** Relative expression of KLF8 protein in SCC9 cells was tested by western blot. **b** The viability of SCC9 cells was assessed by MTT assay. **c**, **d** The migration and invasion of SCC9 cells was measured using wound healing assay and transwell assay, respectively. **e** Relative expression of MMP-2 and MMP-9 protein in SCC9 cells was examined using western blot. **p < 0.01, ***p < 0.001, versus to NC inhibitor; ^&^p < 0.05, ^&&^p < 0.01, ^&&&^p < 0.001, versus to miR-135a-5p inhibitor + si-NC

Next, we further elucidated whether KLF8 was responsible for the regulatory function of DANCR/miR-135a-5p in SCC9 cells using its specific siRNA. Expectedly, miR-135a-5p inhibitor-induced increase of KLF8 was suppressed by the siRNA of KLF8 itself (Fig. 6a). Knockdown of KLF8 attenuated the effects of miR-135a-5p inhibitor on the proliferation, migration and invasion of SCC9 cells (Fig. 6b–d). Similarly, the indicators for tumor development and progression, MMP-2 and MMP-9 were also inhibited by KLF8 silencing (Fig. 6e), which just proved the alterations of tumor malignancies at molecular level. Collectively, these findings demonstrate that KLF8 is responsible for the regulation of DANCR/miR-135a-5p on TSCC progression.

### DANCR knockdown blocked the tumor formation in vivo involving KLF8 activation

**Fig. 7:**
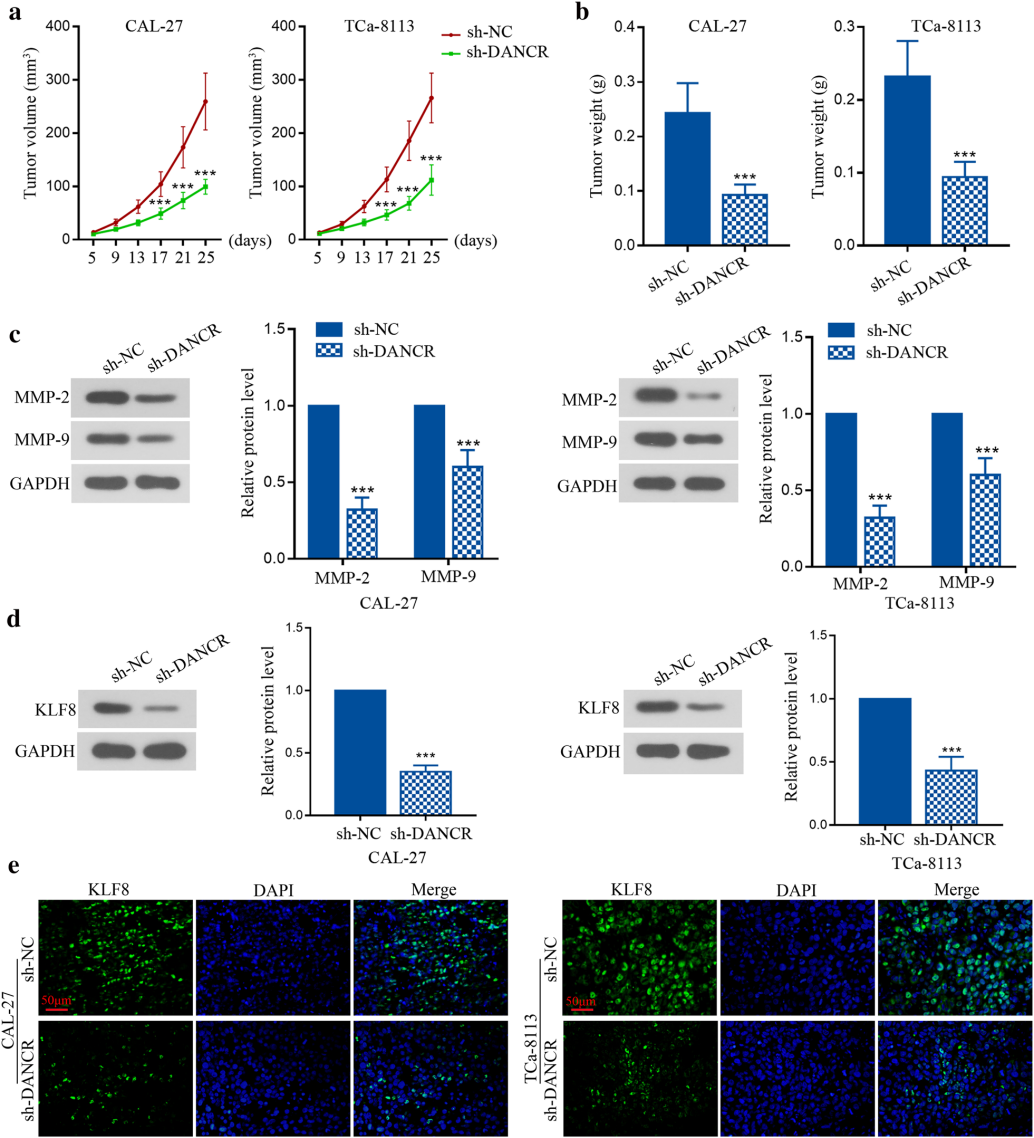
DANCR knockdown blocked the tumor formation in vivo involving KLF8 activation. CAL-27 or TCa-8113 cells transfected with shRNA against DANCR were inoculated subcutaneously into the nude mice. Xenografts were measured every 4 days with a caliper. **a** Tumor volumes were measured every 4 days. **b** Mice were sacrificed after 25 days, and xenograft tumors were excised and weighed. **c** Relative expression of MMP-2 and MMP-9 protein in tumor tissues was measured by western blot after 25 days. **d** Relative expression of KLF8 protein in tumor tissues was examined by western blot after 25 days. **e** Immunofluorescence staining was performed to investigate KLF8 immunoreactive materials in tumor tissues after 25 days. ***p < 0.001, versus to sh-NC

To test the role of DANCR in tumor growth in vivo, CAL-27 or TCa-8113 cells were stably transfected with shRNAs and injected subcutaneously into the right flank of axilla of nude mice. As shown in Fig. 7a, b, it showed that the tumor size and weight could be suppressed by knockdown of DANCR. At molecular level, the expression of MMP-2 and MMP-9 in tumor tissues was also reduced by DANCR inhibition (Fig. 7c). In addition, as shown in Fig. 7d, e, both western blot and immunofluorescence staining demonstrated that a remarkable downregulation of KLF8 was induced in tumor tissues stably transfected with DANCR shRNA. Overall, these in vivo results show that DANCR may activate the expression of KLF8 and MMPs to affect TSCC tumor growth.

## Discussion

Increasing lncRNAs have been revealed to be implicated in the development and progression of various cancers, including TSCC [7, 8, 22]. In this work, DANCR was showed to act as an oncogenic gene to facilitate the proliferation, migration and invasion of TSCC cells through the loss or gain of DANCR. Furthermore, miR-135a-5p was demonstrated to be complementary with DANCR and negatively regulated by DANCR. Overexpression of miR-135a-5p prevented the malignant phenotypes of TSCC cells and reduced the expression of KLF8. Inhibition of miR-135a-5p mediated the protective effects of DANCR silence on TSCC cells. KLF8 was responsible for the regulatory role of miR-135a-5p through modulating MMP-2/9 expression.

Previous reports showed that lncRNA DANCR was high-expressed in esophageal cancer [23], liver cancer [10], colorectal cancer [24], prostate cancer [11], retinoblastoma [25] and so on, which indicated its potential correlation with the poor prognosis of patients. Evidence demonstrated that DANCR enhanced the migration and invasion of prostate cancer cells or gastric cancer cells through impeding TIMP2/3 expression [11] or lncRNA-LET [26]. Jiang et al. suggested that the initiation and progression of osteosarcoma was affected by DANCR via competitively binding to miR-33a-5p [27]. In NSCLC cells, DANCR was found to target miR-758-3p to regulate cell proliferation, migration and invasion [28]. However, up to now, the functional significance of DANCR in the progression of TSCC still requires to be clarified. In this study, the gain- and loss-of-function experiments showed that DANCR could enhance the proliferation, migration and invasion of TSCC cells. The in vivo results further demonstrated that inhibition of DANCR prevented the tumor growth, which indicates the oncogenic role of DANCR in TSCC tumorigenesis.

To the best of our knowledge, this was the first report about the role of DANCR in the progression of TSCC. Emerging references suggested that lncRNAs might function as “sponge” of miRNAs to participate in multiple biological processes. For instance, lncRNA ZFAS1 activated the expression of ZEB1, MMP-14 and MMP-16 to promote tumor growth and metastasis by sponging miR-150 in hepatocellular carcinoma [29]. Wang et al. reported that DANCR facilitated ROCK1-mediated malignant biological behaviors through decoying both miR-335-5p and miR-1972 in osteosarcoma [30]. In this current study, functional experiments indicated that miR-135a-5p overexpression protected against the proliferation, migration and invasion of TSCC cells in vitro, which was showed to directly target DANCR. The inhibitory effects of DANCR silence on TSCC progress could be rescued by silencing miR-135a-5p. Altogether, this study shows that miR-135a-5p serves as a “sponge” miRNA of DANCR to prevent the progression of TSCC.

MiRNAs modulate gene transcription and expression by directly targeting the 3′ UTR of mRNAs, and lncRNAs may exhibit sponging effects on miRNAs during tumor progression. DANCR had been described to competitively bind miR-149 to positively regulate MSI2 expression and promote tumor malignant phenotypes in the pathogenesis of bladder cancer [31]. Although KLF8 expression was altered by DANCR and miR-135a-5p, whether KLF8 was the downstream effector of DANCR/miR-135a-5p to mediate the regulation of TSCC progression was not well understood. Knockdown of KLF8 attenuated the effect of miR-135a-5p inhibitor on TSCC cell proliferation, migration and invasion. More importantly, KLF8 was reported to be a direct target of miR-135a-5p to inhibit NSCLC cell migration, invasion and EMT process by Shi et al. [16]. Together, these results suggest that DANCR/miR-135a-5p axis affects the malignancies of TSCC by the regulation of KLF8.

In addition, MMP is a classical zinc-dependent endopeptidase to affect cell proliferation, angiogenesis, and tumor invasion and metastasis through the degradation of extracellular matrix [32, 33]. MMP-2 and MMP-9 had been demonstrated to be important prognostic biomarkers in diverse cancers, such as breast cancer, colorectal cancer, and NSCLC [34–36]. Considering that KLF8 was highlighted to bind the promoter of MMP-9 to induce its expression and stimulate cancer invasion [37, 38], thus we further examined the alterations of MMPs in the downstream of KLF8. Our data showed that the expression of MMP-9 and MMP-2 was altered by DANCR/miR-135a-5p/KLF8 axis, which just further proved the regulatory network on tumor malignancies from the point of molecular level. Therefore, we conclude that DANCR serves as a “sponge” of miR-135a-5p to activate KLF8/MMP-2/9 signaling pathway, thus stimulating the development and progression of TSCC.

## Conclusion

In conclusion, this present study develops a novel insight that the TSCC tumor progression may be regulated by DANCR/miR-135a-5p/KLF8 axis. To the best of our knowledge, DANCR is suggested to function as a diagnostic biomarker of TSCC for the first time, which may provide new therapeutic targets for the prevention and treatment of TSCC.

## Data Availability

Not applicable.

## Abbreviations

TSCC: tongue squamous cell carcinoma
HNSCC: head and neck squamous cell carcinoma
KLF8: Kruppel-like Factor 8
DANCR: differentiation antagonizing non-protein coding RNA
ceRNAs: competing endogenous RNAs
MMP: matrix metalloproteinase
